# Adulteration of Veratrine with Lime

**Published:** 1845-10

**Authors:** 


*Adulteration of Veratrine with Lime.*—According to M. Versmann, the Veratrine of commerce frequently contains lime, resulting from the employment of lime in its preparation. A small quantity of lime, moreover, facilitates the drying, and renders the preparation more beautiful. It is easily detected on incineration, and is best removed by dissolving the veratrine in spirit, precipitating with sulphuric acid, filtration, driving off the alcohol, and precipitating the veratrine with ammonia.—*Lond. and Ed. Month. Journ. from Buch. Rept.*

